# Understanding the Perspectives and Experiences of Male Perpetrators of Sexual Violence Against Women: A Scoping Review and Thematic Synthesis

**DOI:** 10.1177/15248380241241014

**Published:** 2024-03-28

**Authors:** Josefina Jiménez Aceves, Laura Tarzia

**Affiliations:** 1 The University of Melbourne, Carlton, VIC, Australia; 2 The Royal Women’s Hospital, Parkville, VIC, Australia

**Keywords:** male perpetrators, perspectives, sexual violence, rape, sexual assault, qualitative

## Abstract

Worldwide, sexual violence is a significant public health issue. Although any person can be victimized, the vast majority of sexual violence is perpetrated by men against women. Research has increasingly explored the experiences of victims, however, the perspectives of male perpetrators of sexual violence have largely been sidelined. This limits the ability to design effective public health and policy responses to sexual violence. Our aim was to synthesize the available peer-reviewed qualitative research exploring the perspectives of adult male perpetrators of sexual violence against women. Five databases were searched: MEDLINE, EMBASE, PsychINFO, CINAHL and SocINDEX. We included qualitative, peer-reviewed English-language studies published in the past 40 years, focused on the perceptions and experiences of male perpetrators of sexual violence. Fourteen articles (describing 12 studies) were identified. Most (10) of the articles examined the perspectives of convicted male sex offenders serving a custodial sentence. Of the remaining four articles, two focused on anonymous users of the online forum, Reddit.com, and the remaining two focused on students on university campuses. These four articles were the most recent. We developed four major themes from our thematic analysis of the study findings that represent the experiences and perceptions of male perpetrators of sexual violence. These themes describe deflecting blame onto the victim, external circumstances as mitigating factors, or the perpetrator’s uncontrollable biological urges. One theme involved some expression of remorse or acceptance of responsibility. Although our findings may have implications for prevention and rehabilitation programs, further research is urgently needed in this area.

## Introduction

Sexual violence is a significant health and welfare issue worldwide (World Health Organization, 2013). It is highly prevalent, affecting at least one in every three women globally (World Health Organization, 2013), with proportions increasing in many countries, such as China and Lithuania (Borumandnia et al., 2020). Broadly, sexual violence is defined as any sexual act directed at a person without their consent (World Health Organization, 2013). It may include sexual coercion, sex with a person who is asleep or unconscious, non-consensual choking or rough sex, as well as rape and sexual assault with physical violence (Dartnall & Jewkes, 2013). The activities which constitute sexual violence are often described as existing on a continuum (Koss & Oros, 1982), parts of which—concerningly—can be perceived as “normal” and socially acceptable (Tamborra et al., 2014). This is particularly problematic given that sexual violence is uniquely destructive to the health and well-being of those affected by it (Dworkin et al., 2023), with long-lasting mental health impacts such as anxiety, depression, and post-traumatic stress frequently reported in the literature (Dworkin, 2020).

Most sexual violence is perpetrated by men against women (World Health Organization, 2013, 2021). Indeed, of women who have experienced sexual violence in Australia, for example, more than 99% experienced sexual violence at the hands of a male perpetrator, usually one who was known to them (ABS, 2023). In many cases, the perpetrator was a young man aged between 15 and 30 (Flood et al., 2022). Similar findings are reported elsewhere in the world (World Health Organization, 2013).

In light of this, it is in some ways surprising that, to date, qualitative research on sexual violence has paid little attention to male perpetrators—in particular, men who perpetrate sexual violence against adult women. The overwhelming focus of the existing literature has been on identifying and responding to the accounts of female victims of sexual violence (Lea & Auburn, 2001; O’Sullivan, 2005; Tarzia et al., 2020).^1^ In practical terms, this means that what we know about the male perpetrators of sexual violence is primarily derived by researchers from the accounts and perceptions of female victims (Easteal & McOrmond-Plummer, 2006; Parkinson, 2017). Our understanding of the perspectives, motives and justifications of male perpetrators is thus usually second-hand at best.

Of course, it is appropriate that women victims be a central focus of sexual violence research (Scully, 1990). A research focus on female victims has foregrounded women’s marginalized voices (Jeffrey & Barata, 2018). It has been critical in dispelling harmful stereotypes and developing appropriately tailored support for victims of sexual violence (Tarzia et al., 2017). Further, research focusing on victims may be empowering for individual research participants (Valpied et al., 2014), and sends a validating message to victims more broadly. Nonetheless, it would be a disservice to women if there was not also some corresponding focus on directly understanding the men who perpetrate sexual violence (Wood, 2004). As Scully (1990) among others, has observed, focusing overwhelmingly on victimized women may unintentionally encourage a perception that sexual violence is a women’s rather than a men’s problem (Anderson et al., 2021). It may also incorrectly place the emphasis on a female victim’s role in an encounter with a male perpetrator (O’Sullivan, 2005). As Doyle (2012, p. 17) has recently observed:

I know the desire to be victim-focused comes from a good place, but I can’t help wondering why the response ought not be perpetrator-focused. Surely, we want to stop them doing what they do? Isn’t that more important? Or, at least, doesn’t that come first?

Directly understanding the perspectives of male perpetrators is imperative to efforts to prevent male-perpetrated sexual violence and to protect women from harm. At the societal level, if the myths and discourses which appeal to and embolden male perpetrators are to be challenged, they must be first identified and understood (Lea & Auburn, 2001; Scully, 1990). Likewise, if the structural and cultural factors that incentivize men to perpetrate sexual violence are to be withdrawn, they must first be located in the accounts and attitudes of men (Muchoki, 2011; Scully & Marolla, 1985). To ignore male perpetrators is to allow a culture of male impunity to continue.

Likewise, at the individual level, prevention and intervention programs targeting potential or actual male perpetrators of sexual violence will be more effective the better the perspectives and lived experiences of the target cohort are understood (Tarzia et al., 2020). Similarly, a more nuanced understanding of the differences in the beliefs and motivations among male perpetrators can allow for more tailored and, therefore, more effective interventions (Beech et al., 2006). This has been recognized in some recent policy responses to violence against women, which have emphasized keeping “perpetrators in view” and involved initiatives designed to improve our understanding of perpetrators and the effectiveness of existing interventions (Commonwealth of Australia, 2022).

With that in mind, we conducted a preliminary scoping review seeking to identify what it is we currently know about the perspectives of male perpetrators of sexual violence from male perpetrators themselves. “Perspectives” is a broad category encompassing a perpetrator’s lived experiences, including their narratives, discourse, understandings, reasons, motives, and rationalizations. To our knowledge, this is the first review to synthesize the qualitative literature on this topic. In conducting the scoping review, we sought to identify and map the existing peer-reviewed literature, as well as identifying gaps for further research.

## Method

A scoping review is a relatively new form of literature review (Brien et al., 2010). It is commonly known as a “mapping review” (Peters et al., 2015) and is an increasingly popular approach to reviewing health research evidence (Levac et al., 2010). In broad terms, the purpose of a scoping review is to identify the current state of understanding in a particular research area (Anderson et al., 2008). A scoping review seeks to rapidly map the key concepts, sources, and types of evidence with respect to a particular research area, as well as identifying areas where the research literature is sparse or lacking (Arksey & O’Malley, 2005). Commonly, scoping reviews are a preliminary form of review, used to determine the feasibility of, and to usefully inform, future systematic reviews (Arksey & O’Malley, 2005; Grant & Booth, 2009). However, they may also be usefully undertaken as a stand-alone study. That is especially so where, as here, the research area is complex or has not been previously reviewed comprehensively (Mays et al., 2001). Scoping reviews do not require that the literature in a particular area be assessed for quality, meaning they are also comparatively time efficient (Arksey & O’Malley, 2005).

### Search Strategy

Following a series of consultations with a librarian and the testing of several provisional search strategies, five databases were ultimately chosen for the search: MEDLINE, EMBASE, PsychINFO, CINAHL and SocINDEX with full text. A parallel search of Google Scholar was conducted for additional peer-reviewed literature, although this did not yield any additional results. Searches for relevant studies were conducted using these databases in September 2022. Searches of MEDLINE, EMBASE and PsychINFO were conducted through OVID, whereas searches of CINAHL complete and SocINDEX with full text were conducted through EBSCO.

Prior testing of provisional search strategies suggested that the relevant literature was limited and scattered across different fields of research. This indicated the need to employ broad search terms and an inclusive search strategy to elicit a group of records that could confidently be thought to contain the relevant literature. In line with this, a combination of several groupings of terms was used. The first grouping of alternative terms was designed to elicit a primary pool of studies, the subject of which was sexual violence of some sort. A second grouping of alternative terms was designed to narrow that pool to studies which had some focus on perpetrators. A third grouping of alternative terms was designed to further narrow the pool to those which had a focus on the perspectives of perpetrators. A fourth grouping of alternative terms was designed to further restrict the pool to studies which employed a qualitative or at least a mixed methodology. A final grouping of alternative terms was designed to exclude from the remaining records irrelevant literature commonly seen in the preliminary testing. In particular, these terms sought to filter out literature relating to: female rather than male perpetrators; the perspectives of victims of sexual violence and bystanders rather than perpetrators; violence other than sexual violence; and sexual violence by or against children rather than adults. An example of a full search strategy (including search terms and their combinations) is shown in Table 1. Variations of this search strategy were used with all databases following necessary adjustments to truncations, wildcards, and Boolean operators.

**Table 1.** Example Search Strategy.

1	“sexual violence” or “sexual abuse” or “sex* offen*” or “rape” or “sexual assault” or “sexual coercion”
2	ipsv or “intimate partner sexual violence”
3	perpetrator* or abuser* or offender*
4	“rapist*” or “sex offender*” or “sex abuser*” or “sexual abuser*” or “sexual predator*”
5	1 or 2 adj4 3
6	4 or 5
7	perspective* or view* or perception* or attitude* or opinion* or understanding or experience* or discourse or talk
8	qualitative or survey or interview* or oral or “focus group*”
9	reason* or motivation* or incentive**
10	7 or 8 or 9
11	6 and 10
12	“female perpetrator*” or ((women* or victim* or female* or survivor* or bystander*) adj3 (perspectiv* or view* or attitud* or narrativ* or percept* or opinion* or experience* or ‘lived experience*’ or account*)) or battery or “child abuse”
13	11 not 12

### Eligibility Criteria

As the focus was on the perceptions of perpetrators, only qualitative studies were included. Studies were included if the participants were mainly adult male perpetrators of sexual violence, including rape, sexual assault, and sexual coercion. Studies of female and mainly juvenile perpetrators were excluded. Studies were included if the participants had perpetrated sexual violence mainly against adult women. On the other hand, studies mainly focused on perpetrators of sexual violence against men or against juveniles of any gender were excluded.

Studies focused on the subjective perceptions of the perpetrator cohort about their offending in any relevant respect were included—for example, studies examining participants’ perspectives, views and attitudes toward their sexual violence or their reasons and justifications for committing sexual violence. On the other hand, we excluded studies examining participants’ perspectives on other matters, such as their perspectives on the criminal justice or prison systems or their experience of post-offense intervention programs or other interventions. We also excluded studies focusing on the perspectives of non-perpetrators who are in some way implicated, affected, or involved in the perpetration of sexual violence. This excluded, for example, studies focusing on the perspectives and attitudes of victims and their families, policymakers, actors in the criminal justice and health systems, and bystanders.

Peer-reviewed journal articles were included, while conference abstracts, theses, books, and book chapters were excluded. Additional peer-reviewed publications, such as research or government reports, were considered for inclusion, although ultimately, none were identified. Studies of the requisite type were included, provided they were published in the last 40 years, that is, on or after 1982. This date range was selected in part because provisional search strategy testing suggested that there was little if any, relevant literature before this time. Furthermore, we wished to develop an understanding of perpetration in social contexts that were similar to the present day. Studies were excluded if they were not written in English.

### Data collection and Extraction

The search strategy identified 5,113 records for screening. Following the removal of 582 duplicates, 4,529 records remained for screening. We used the Covidence platform (https://www.covidence.org) for screening. Covidence is a web-based platform designed to expedite and facilitate the systematic review of records and to identify and resolve disagreements, if any, between reviewers. The records extracted from the databases were uploaded to the platform for review.

**Figure 1.**
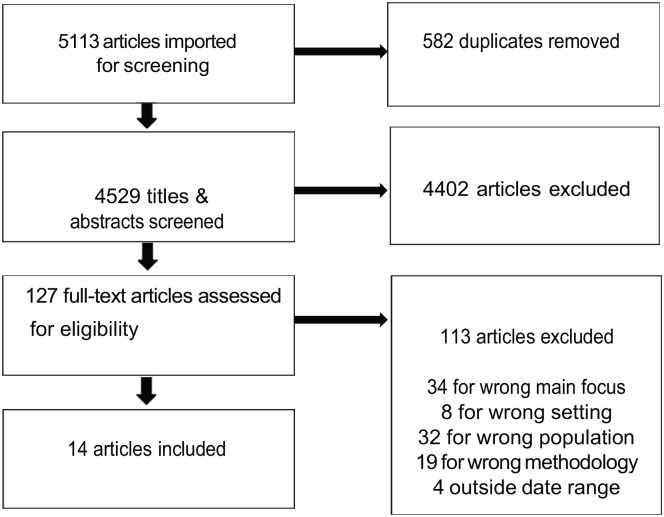
Screening and study selection process.

The first author screened the 4,529 titles and abstracts of the citations retrieved and rejected those which did not meet the inclusion criteria or triggered one or more exclusion criteria. Four thousand four hundred two studies were rejected at this first stage of screening. The first author then conducted a full-text review of the remaining 127 studies to approve eligibility, with input from, and ongoing discussion with, the second author. Ultimately, a further 113 studies were excluded at the stage of full-text screening, leaving 14 included articles (comprising 12 studies). Our screening and study selection process is shown in Figure 1.

The studies excluded during screening were typically excluded for one or more of the following reasons:

- they focused on the perspectives and lived experiences of victims or other actors involved in or affected by sexual violence, such as medical professionals, participants in the criminal justice system, policymakers, or particular demographic groupings;
- they focused on non-adult perpetrators, or more commonly, non-adult victims of sexual violence;
- they focused on the perpetration of sexual violence in highly specific and non-transferrable contexts, such as wartime sexual violence;
- they focused on the perspectives of perpetrators about their participation in the criminal justice system or post-offending interventions and treatments rather than the experience of perpetration.

Study characteristics, findings, and recommendations were inputted into a customized table (Table 2). Themes were developed in an iterative fashion using Braun and Clarke’s reflexive thematic analysis (Braun & Clarke, 2006; Clarke & Braun, 2018). We used Lumivero’s NVivo platform to code and re-code the articles.

**Table 2.** Characteristics of Included Studies.

Author/Year/Location	Research Aim	Study Participants and Methods	Main Findings
Beech et al. (2006), United Kingdom.	To determine whether implicit theories (“dangerous world”; “women as sexual objects”; “entitlement”; “male sex drive is uncontrollable”; and “women are dangerous”) reported in other research could be identified in a British sample of rapists, and, if so, whether this could inform treatment provision for rapists.	*N* = 41. Convicted rapists serving custodial sentences and participating in prison sex offender treatment programs. Mixed ethnicity (Caucasian, African, Asian and mixed race) with an average age of 33.6 years. Semi-structured interviews. Qualitative analysis.	The implicit theories were identified in the British sample of rapists, with the “dangerous world” and “women as sexual objects” implicit theories most prevalent. The distribution of theories suggested three groups of rapists: a group with violent motivations; a group with sexual motivations; and a sexually sadistic group with both violent and sexual motivations. This typology has implications for tailoring treatment to offender motivations.
Blagden et al. (2014), United Kingdom.	To explore the experiences and perspectives of sexual offenders in denial about their offending and to identify implications for a treatment paradigm which typically views denial as a barrier to constructive treatment.	*N* = 10. Convicted sexual offenders serving custodial sentences and categorically denying offending. Semi-structured interviews and repertory grid interviews. Qualitative (interpretative phenomenological analysis).	Two broad themes emerged. First, participants rejected and sought to distance themselves from the label of sexual offender and associated stigma. Secondly, participants exhibited common modes of thinking, including grievance thinking, narratives of temporal consistency of identity, and constrictive thinking. This suggested that offenders in denial may be more amenable to treatment than commonly thought.
Brennan et al. (2018), United States.	To understand sexual assault perpetrators’ emotional responses to perpetration and, in particular, to examine men’s narratives of sexual perpetration against women. This is to facilitate improvements in treatment and other programs designed to prevent repeat perpetration.	*N* = 61. Anonymous users of the Reddit online community, likely primarily men aged 18–39. Narrative responses to a question posed to a Reddit forum. Qualitative (text analysis with elements of phenomenological design and discursive analysis)	Four primary emotional responses to perpetration emerged: shame, guilt, anger, and depression. Of these, guilt appeared to be the most adaptive response for protecting against repeat perpetration.
Dumbili and Williams (2020), Nigeria.	Explore how Nigerian sociocultural constructions of alcohol consumption facilitate sexual violence against women in a Nigerian university.	*N* = 31. 22 male and 9 female undergraduate students at a Nigerian university, aged 19–23 years old. All but one a member of a single ethnic group (Igbo). Semi-structured interviews. Qualitative (thematic coding).	Alcohol consumption is gendered, with women confined to drinking sweetened alcoholic beverages with a higher alcohol content. Further, alcohol consumption facilitates sexual violence as some men buy such drinks for women, pressure them to excessively consume them, and sexually assault them once inebriated. In this way, this practice contributes to the risk of attempted and completed rapes on campus.
Hipp et al. (2017), United States.	To study first-person narratives of sexual assault perpetration and perpetrators’ own descriptions of, and justifications for, sexual assault.	*N* = 68. Anonymous users of the Reddit online community, likely primarily men aged 18–39. Narrative responses to a question posed to a Reddit forum. Qualitative analysis (thematic coding).	Several themes related to perpetrators’ justifications for sexual assault emerged: sexual scripts; victim blame; hostile sexism; biological essentialism; objectification of women; and socio- sexuality. The justifications showed reliance on sexual scripts that contribute to victim blaming and objectification of and hostility toward women. The findings suggest that direct intervention with perpetrators is necessary, and an element that must be considered is the way in which social structures contribute to and legitimize these behaviors.
Jeffrey and Barata (2018), Canada.	To examine sexual violence in the relational context and from men’s perspectives, including how they talk about and frame their behavior. To examine how men’s sexual violence and accounts reflected and enacted the normalization of violent heterosexuality.	*N* = 10. Male university students aged 18–22 who reported engaging in sexual violence of some kind (mainly verbal coercion and taking advantage of victims’ intoxication). Eight of the ten identified as heterosexual. Seven of the ten identified as White/European (two as Southeast Asian and one as Caribbean/White). Screening survey followed by individual in-depth interviews. Qualitative (discourse analysis)	Male perpetrators of sexual violence often used language that helped them to position themselves and their sexual violence as normal and expected. They also used alternative discourses and accounts about sexual violence, heterosexuality, and consent. These alternative (and sometimes contradictory) discourses involved the perpetrators positioning their behavior as wrong and consequential. The discourse identified has implications for the design of future educational and intervention campaigns.
Lea and Auburn (2001), United Kingdom.	Explore the rape narratives of a convicted rapist, the ways in which he accounts for his crime, and the practical ideologies on which he draws in describing and explaining his actions.	*N* = 1. A convicted rapist serving a custodial sentence and participating in group therapy sessions in a sex offender treatment program. Transcripts of group therapy sessions. Qualitative (discourse and conversation analysis).	The analysis identified two main “practical ideologies” that the subject used to account for his interaction with his victim: coercion (which is associated with rape) and mutuality (which is associated with consensual sex). The interplay of these discourses creates ambiguity, blurring the line between rape and sex and downgrading the perpetrator’s responsibility. This has therapeutic implications, including as to the use of language in treatment programs.
Murphy and Winder (2016), United Kingdom.	To increase understanding of adult male sex offenders who perpetrate sexual violence against the elderly, that is, victims aged 55 and over.	*N* = 5. Convicted sex offenders (whose victims were aged 55 or over), serving a custodial sentence. Semi-structured interviews. Qualitative analysis (interpretative phenomenological analysis.)	Four key themes emerged for making sense of their behavior: (a) “Life’s been really tough”; (b) “I’m not bad, I did what anyone would do”; (c) “Other people haven’t helped me or have made things worse”; and (d) “Coping and pleasure.” Accounts focused on external factors and downplayed personal choice and sexual desire. While there was some acknowledgment of wrongdoing, there was not full acceptance of responsibility.
Polaschek et al. (2001), New Zealand.	To develop a descriptive model of the offense process based on the narrative accounts of men who had sexually assaulted adults.	*N* = 24. Convicted male rapists (of a victim aged over 16), serving a custodial sentence. Oral narrative of offending obtained by semi-structured interviews.	A six-stage descriptive model identified, comprising (a) background factors to the offense; (b) goal formation; (c) approach behavior, (d) offense preparation; (e) the offense; and (f) post-offense behavior.
Polaschek and Gannon (2004), New Zealand.	To determine whether implicit theories (“dangerous world”; “women as sexual objects”; “entitlement”; “male sex drive is uncontrollable”; and “women are dangerous”) reported in other research could be identified in the offense process descriptions of incarcerated sex offenders, and if so, to what extent.	*N* = 37. Convicted male sex offenders, serving a custodial sentence. Interviews, open-ended questions. Qualitative analysis.	The relevant implicit theories were present in the offense process descriptions. The three most prevalent implicit theories were: (a) women are dangerous; (b) women are sex objects; and (c) entitlement. The other implicit theories—(d) male sex drive is uncontrollable and (e) dangerous world —occurred in a small minority. The existing implicit theories were sufficient.
Scully and Marolla (1984), USA.	To analyse the excuses and justifications of convicted and incarcerated rapists and their perceptions of their offending.	*N* = 114. Convicted male rapists (or attempted rapists), serving a custodial sentence. Interview involving open-ended questioning. Qualitative analysis.	All subjects included excuses and justifications in their accounts of offending. Rape admitters believed that rape was morally reprehensible, but appealed to forces outside their control (alcohol, drugs), rape stereotypes and victim blaming, allowing the majority to view themselves as non- rapists or ex-rapists. Rape deniers had no compelling reason not to rape (a form of entitlement) and justified their behavior as appropriate in the situation. They denied through cultural rape stereotypes such as victim blame. Strong indications that rapists are motivated by cultural perspectives rather than idiosyncratic illness.
Scully and Marolla (1985), USA.	To attempt to examine rape from the perspective of convicted and incarcerated rapists, and to identify the function of sexual violence in their lives, and the perceived rewards for them in raping.	*N* = 114. Convicted male rapists (or attempted rapists), serving a custodial sentence. Interview involving open-ended questioning. Qualitative analysis.	Rape is frequently a: (a) means of a revenge and punishment, including punishing women generally; (b) fantasy or particularly exciting form of dominant and controlling impersonal sex. Women were seen as dehumanized sexual commodities, and rape was viewed as a high-reward low- risk act. These perspectives point to rewards and cultural supports for rape, not just individual pathology.
Scully (1988), USA.	Explore the image of self and victim held by a group of convicted rapists to determine the social factors that contributed to the failure of individual self-controls.	*N* = 79. Convicted male rapists serving a custodial sentence. Interview involving open-ended questioning. Qualitative analysis.	The role taking for admitters (men who define their behavior as rape) was that they lacked self-control. They seem to know what they were doing while they did it, but their response was concern for their own well-being or indifference. While they perceived themselves as violent and subhuman and believed they terrified their victims, they took satisfaction from humiliating and degrading them. The majority of admitters expressed regret and sorrows for the victim during the interviews. The role taking in deniers (men who do not define their behavior as rape) was relatively absent of reflection. They didn’t care how the victim perceived them, or they believed she liked them. They were unaware of the victim’s feelings or thought she eventually enjoyed the rape. They expressed no emotion or anger or concern for their well-being. For both groups, women were perceived as worthless sexual objects.
Weldon (2015) Scotland.	Explore cognitions present in intimate partner violence sex offenders, representative of their view of themselves, others, and the world.	*N* = 11. Convicted male sex offenders, serving a custodial sentence. Interviews semi- structured and non- prescriptive. Qualitative analysis (interpretative phenomenological analysis).	Fourteen themes thought to be representative of implicit theories in intimate partner violence sex offenders emerged: These are: violence is acceptable; grievance/revenge; dangerous world; need for control; real man; entitlement/women are objects; male sex drive/policing partner; women are provoking; rejection/abandonment; women are supportive; uncontrollability; nature of harm; the new me; I am not like them. Of these, four themes (rejection/abandonment, women as supportive, need of control, and real man) seem to be explicit to intimate partner violence sex offenders.

The first author is a doctoral student with a background in human development, mindfulness, mediation, and self-compassion. The second author has a background in sociology and has been researching in the field of sexual and intimate partner violence for over a decade, including working with men who have used violence. Both authors bring a feminist lens to the problem of sexual violence and acknowledge the complexity of the issue and the role of social structures in shaping violence-supportive attitudes. We mention our backgrounds because we acknowledge that they are a potential influence on the thematic analysis.

## Results

### Descriptive Summary

There were 14 articles included in this review, representing 12 studies. This is because three articles (Scully, 1988; Scully & Marolla, 1984, 1985) used the same study sample. A further two studies had overlapping cohorts (Polaschek & Gannon, 2004; Polaschek et al., 2001). Another two studies (Brennan et al., 2018; Hipp et al., 2017) analyzed a slightly differently constituted sample of responses to the same online thread, making it likely that there is at least some overlap between those study cohorts.

The study sample sizes ranged from one participant (Lea & Auburn, 2001) to 114 participants (Scully & Marolla, 1984, 1985). The studies spanned the 1980s (Scully & Marolla, 1984) to 2020 (Dumbili & Williams, 2020). Aggregating the size of the study cohorts is revealing. Across all of the studies, over the past 40 years, researchers have directly engaged with the perspectives of no more than around 400 male perpetrators.

In most of the studies (*n* = 8) the participants were convicted male sex offenders serving a custodial sentence (Beech et al., 2006; Blagden et al., 2014; Lea & Auburn, 2001; Murphy & Winder, 2016; Polaschek & Gannon, 2004; Polaschek et al., 2001; Scully, 1988; Scully & Marolla, 1984, 1985; Weldon, 2015). In most cases, the participants in these studies had been convicted of rape or some other serious sexual offense, and often multiple such offenses. In many cases, these participants were also participants in some form of sex offender treatment program delivered in custody. In the studies involving perpetrators not convicted of offenses, the perpetrators self-disclosed their perpetration of sexual violence.

The subjects of two studies were anonymous users of an online forum, Reddit.com (Brennan et al., 2018; Hipp et al., 2017), and the subjects of the remaining two studies were students on university campuses (Dumbili & Williams, 2020; Jeffrey & Barata, 2018). These four studies were the most recent, and they canvassed a wider range of sexual violence behaviors than the studies of incarcerated offenders, including more subtle forms of sexual violence such as verbal coercion and alcohol-facilitated sexual violence. However, in general, the studies confirmed observations elsewhere in the literature that the relevant research tends to be focused on convicted and incarcerated serious sexual offenders, with comparatively little focus on sexual violence in its more insidious and “normalized” forms (Jeffrey & Barata, 2018).

Geographically, the studies were concentrated in the “Global North.” Three of the included studies were conducted in the United States (Brennan et al., 2018; Hipp et al., 2017; Scully, 1988; Scully & Marolla, 1984, 1985) and one in Canada (Jeffrey & Barata, 2018). However, two of the studies conducted in the United States (Brennan et al., 2018; Hipp et al., 2017) involved anonymous users of Reddit, a U.S.-based English-language online forum, at least some of whom were likely located elsewhere. Five studies were conducted in the United Kingdom (Beech et al., 2006; Blagden et al., 2014; Lea & Auburn, 2001; Murphy & Winder, 2016; Weldon, 2015). Two studies were conducted in New Zealand (Polaschek & Gannon, 2004; Polaschek et al., 2001). One study was conducted in Nigeria (Dumbili & Williams, 2020).

Most of the studies (*n* = 9) collected data from the participants via one or more semi-structured interviews, often preceded by a screening questionnaire or similar instrument. In the three studies where participants were not interviewed, some form of textual or discourse analysis was applied to the participants’ online responses (Brennan et al., 2018; Hipp et al., 2017) or transcripts of the participant’s contributions to a group therapy session in a prison treatment program (Lea & Auburn, 2001).

All of the studies used some form of qualitative analysis to analyze the data obtained from participants. Most commonly, the studies used thematic analysis or interpretative phenomenological analysis. Sometimes, this was augmented by the use of other analytic techniques, such as discourse analysis. Attributes of the studies are set out in more detail in Table 2.

## Main Findings

This review sought to understand the perceptions of male perpetrators of sexual violence. Four main themes were developed from analysis of the included 14 articles, which we have termed as follows:

• “She deserved it.”
• “I’m a good guy.”
• “I couldn’t stop myself.”
• “I’m really sorry.”

These themes are outlined below in detail, with quotes taken directly from the study participants.

### Theme 1: “She Deserved It”

The “She deserved it” theme was the strongest within the data, represented in all but one of the 14 articles (Blagden et al., 2014). It was coded in participant accounts 60 times. This theme described the perpetrators displacing blame onto the victims regardless of their own actions and exhibiting no regret. It encompassed four sub-themes, which we have termed:

• “She seduced me.”
• “She’s a slut.”
• “She’s a sexual object.”
• “She must be punished.”

We address these sub-themes in the following sections.

#### Sub-Theme 1: “She Seduced Me.”

Many perpetrators blamed the victim. They described the victim as deliberately sexually provocative, thereby inviting the use of sexual violence. For example, various perpetrators accused the victims of “leading them on,” “playing a game” or “seducing” them. The threshold for perceived (or imagined) sexually provocative behavior was typically very low and included the victim being “friendly” (Scully & Marolla, 1984, p. 535); inviting the perpetrator to her home (Brennan et al., 2018; Murphy & Winder, 2016); initiating physical contact (Brennan et al., 2018; Murphy & Winder, 2016); hitchhiking or accepting a ride from the perpetrator (Polaschek & Gannon, 2004; Scully & Marolla, 1984, 1985); going on a date with the perpetrator (Polaschek & Gannon, 2004); failing to resist; wearing “short skirts” (Scully & Marolla, 1984, p. 537); drinking with the perpetrator (Dumbili & Williams, 2020); walking at night (Lea & Auburn, 2001); standing at a window (Beech et al., 2006); speaking frankly about previous sexual experiences (Hipp et al., 2017), and having previously had sex with the perpetrator (Brennan et al., 2018). For example:

[By discussing sex] it was like she was saying, “rape me.” (Scully & Marolla, 1984, p. 535).

She starts to get undressed, she’s obviously conceded. (Polaschek et al., 2001, p. 532).

I don’t believe in date rape. If she puts herself in that position . . . (Polaschek & Gannon, 2004, p. 307).

Yes, I’ve done it and I do not regret it. She led me on all night. (Hipp et al., 2017, p. 87).

#### Sub-Theme Two: “She’s A Slut.”

Under this sub-theme, perpetrators labeled the victim as sexually promiscuous, a “slut” or a “whore,” whom they felt entitled to have sex with at will. In contrast to the previous theme, there was no attempt to identify any specific sexual provocation aside from the victim’s status as a “slut” or equivalent. Again, the threshold for perceived (or perhaps more accurately, imagined) sexual promiscuity was set very low. For example:

If she’s a bit tipsy, you will know the kind of person she is. You’ll know if she is the kind of person you’ll just “run” [have sex with] straightaway (Dumbili & Williams, 2020)

To be honest, we . . . knew she was a damn whore and whether she screwed one or 50 guys didn’t matter. (Scully & Marolla, 1984, p. 536).

She was a slut and she suited my purpose, and it was a game of cat and mouse . . . and she lost the game (Polaschek & Gannon, 2004, p. 300).

#### Sub-Theme Three: “She’s A Sexual Object”

In this sub-theme, perpetrators perceived the victim as deserving of sexual violence simply because she was a woman. Women were seen to be subhuman and worthless sexual objects, mere instruments for the sexual desire of the perpetrator, which was the overwhelming priority. They were expected to provide sex under all circumstances, and their consent was irrelevant. In this mode of thinking, the withholding of consent or resistance was not only invalid but an invitation for the perpetrator to try harder and apply more coercion and force. For example:

She said “no” but it was a societal no, she wanted to be coaxed. All women say “no” when they mean “yes” but it’s a societal no, so they won’t have to feel responsible later. (Scully & Marolla, 1984, p. 535).

I had no feelings at all, she was like an object. (Scully, 1988, p. 209).

So I thought, “I’ll have sex instead, I’ll go and rape someone”. (Polaschek et al., 2001, p. 530).

I basically used her as my personal fuck-box. . .she was just dead weight. Basically, I tried to throatfuck her. . . She resisted by just tilting her head out of the way. . .She could barely open her eyes. Then she threw up. . .and fell into a fetal position. . .I just wanted to finish so I started jackhammering. (Hipp et al., 2017, p. 87).

#### Sub-Theme Four: “She Must be Punished.”

In this sub-theme, perpetrators sought to normalize their use of sexual violence by describing it as reasonable punishment, or a way to get even for the things that the victim said or did. Often the trigger for punishment was the victim belittling the perpetrator or “putting him down” (Weldon, 2015, p. 295). For example:

Well, I’m afraid I lost my temper, and just then, just did something wrong really, just lost my temper and couldn’t forget, anger at her, things she was saying and things she did, anger at her . . . brought up a lot of feelings over the 4½ year term of the relationship and fed the anger, where it got to the stage where, I was so angry at her, it was almost as if I wanted to get even. (Beech et al., 2006, p. 1641).

But sometimes, the victim was seen as deserving of punishment on behalf of *other* women that the perpetrator thought had mistreated him. For example, one convicted offender raped and beat his victim after fighting with his wife. He located his victim after driving around “thinking of hurting someone.” He said (Scully & Marolla, 1985, p. 255):

I have never felt that much anger before. If she had resisted, I would have killed her . . . The rape was for revenge. I didn’t have an orgasm. She was there to get my hostile feelings off on.

Another perpetrator expressed similar sentiments (Scully & Marolla, 1985, p. 255):

During the rapes and murders, I would think about my girlfriend. I hated the victims because they probably messed men over. I hated women because they were deceitful, and I was getting revenge for what happened to me.

### Theme Two: I’m A Good Guy

The “I’m a good guy” theme was the next most common theme. It appeared in the accounts of participants in 10 of the 14 articles (Beech et al., 2006; Blagden et al., 2014; Brennan et al., 2018; Hipp et al., 2017; Jeffrey & Barata, 2018; Lea & Auburn, 2001; Murphy & Winder, 2016; Polaschek & Gannon, 2004; Polaschek et al., 2001; Scully, 1988; Scully & Marolla, 1984; Weldon, 2015). In those articles, it was coded in participant accounts 49 times.

In the “I’m a good guy” theme, as in the previous theme, perpetrators frequently sought to displace blame and mitigate their responsibility. However, in contrast with the previous theme, perpetrators did not blame their victims, but rather, external factors. Also, in contrast with the previous theme, perpetrators expressed some level of regret. The perpetrators’ emphasis on contributing external factors and conditions appears to be part of an attempt to disavow any intent and most, if not all, moral responsibility (Scully & Marolla, 1984).

This theme encompasses four sub-themes, which we have termed:

• “It was out of character.”
• “I was intoxicated.”
• “I was a gentleman.”
• “I was going through a tough time.”

#### Sub-Theme One: “It Was Out of Character.”

Under this sub-theme, many perpetrators characterized their sexual violence as unusual, unexpected, and uncharacteristic, and so less problematic than the sexual violence of other men (Weldon, 2015). This appeared to be a form of perpetrator image management. It also appears to serve as a coping mechanism by which perpetrators seek to maintain and protect a positive self-image or identity and avoid identifying as a “real rapist.” Thus, perpetrators could simultaneously admit perpetrating sexual violence while emphasizing that it was out of character. The sexual violence was described as “not part of my personality” (Scully & Marolla, 1984, p. 539) or “not in my nature” (Blagden et al., 2014, p. 1719) or not what the perpetrator is really “like” or the result of being “temporarily sick” (Scully & Marolla, 1984, p. 540). For example:

It’s different from anything else I’ve ever done. I feel more guilt about this. It’s not consistent with me. When I talk about it, it’s like being assaulted myself. I don’t know why I did it, but once I started, I got into it. Armed robbery was a way of life for me, but not rape. I feel like I wasn’t being myself. (Scully & Marolla, 1984, p. 541).

I didn’t want to hurt her. Erm, because I’m not like that. Erm, but at the time, I pulled this knife out. And I, errrr, dragged her onto the ﬂoor. (Murphy & Winder, 2016, p. 806).

#### Sub-Theme Two: “I was Intoxicated.”

Here, perpetrators foregrounded the contributing role of intoxicants. For example, many attributed their offending to the effects of alcohol and drug abuse. One perpetrator suggested he didn’t “have the guts to rape” until consuming eight beers and taking four “hits” of acid (Scully & Marolla, 1984, p. 38). Another perpetrator emphasized that his offending followed thirteen hours of continuous drinking in an unsuccessful attempt to forget his feelings (Murphy & Winder, 2016, p. 811). Other perpetrators described committing sexual violence in a “blackout” or “near blackout” state (Brennan et al., 2018, p. 400) following the consumption of a mix of intoxicants. In such cases, perpetrators often denied memory of much of the incident. One perpetrator stated (Beech et al., 2006, p. 1642):

Well basically, I can’t remember how it came about. I was actually drunk, and I have stated that all the way through. I can remember parts of it at the end, being apologetic, and things like that.

For some perpetrators, intoxication was claimed to be the direct cause of the sexual violence. For such perpetrators, the desire to commit sexual violence was apparently something foreign, introduced by the intoxicants consumed. For example (Murphy & Winder, 2016, p. 807):

[My] old head, started to fantasize about [the victim] and that. Because of my drugs and, thing I was taking. So, that started to enter my head. And so, I used to think about. (Slight pause) I wonder what [the victim] is like in the nude.

For other perpetrators, the contributing role of intoxication was more ambiguous and did not entirely absolve the perpetrator of self-blame. On such accounts, a desire to commit sexual violence was revealed rather than introduced by the use of intoxicants. For example:

[Alcohol and drug use] brought out what was already there but in such intensity it was uncontrollable. Feelings of being dominant, powerful, using someone for my own gratification, it all rose to the surface. (Scully, 1988, p. 538).

I don’t want to use my intoxication as an excuse, but I’ve never done anything like this sober. (Brennan et al., 2018, p. 400).

#### Sub-Theme Three: “I Was A Gentleman”

Other perpetrators sought to maintain a positive self-image by emphasizing that, during the sexual violence, they were nonetheless decent or gentlemanly. It seems that this group sought to maintain a positive self-image not by displacing blame for their sexual violence but by positively distinguishing themselves from other perpetrators. Typically, they sought to demonstrate that during the relevant encounter, they were focused on the victim’s needs, not their own. For example:

I try to make a girl enjoy herself as much as possible and she was no exception. (Scully, 1988, p. 205).

I took my jacket off and laid it down for her to lie on. It’s important to say that because the jacket comes into it in a moment, and she got on the jacket, and we started. . . I started raping her. (Lea & Auburn, 2001, p. 28).

I don’t feel any major pleasure except for the tenth of a second when I’m ejaculating. (Polaschek et al., 2001, p. 535).

#### Sub-Theme Four: “I was Going Through A Tough Time.”

Here, perpetrators emphasized the effects of mental health struggles or other hardships. They pointed, for example, to the influence of a “mental disorder” (Scully & Marolla, 1984, p. 540) or of being in a “dark and horrible place in . . . life” (Brennan et al., 2018, p. 408). For example:

The fact that I’m a rapist makes me different. Rapists aren’t all there. They have problems. It was wrong so there must be a reason why I did it. I must have a problem.” (Scully & Marolla, 1984, p. 539).

### Theme Three: “I Couldn’t Stop Myself”

This was the third most common theme, described in 6 of the 14 articles (Hipp et al., 2017; Jeffrey & Barata, 2018; Murphy & Winder, 2016; Polaschek & Gannon, 2004; Scully, 1988; Scully & Marolla, 1985). In those articles, it was coded in participant accounts 25 times.

In this theme, perpetrators sought to explain their use of sexual violence because of loss of control and powerlessness in the face of uncontrollable sexual urges. Again, this was a mechanism used to minimize personal responsibility. In this case, blame was displaced onto the male biology, which was said to make it virtually impossible to desist from sex past a certain point. For example:

The old [penis] rules the brain. (Polaschek & Gannon, 2004, p. 307).

You know, its life isn’t it . . . a man’s a predator. Not in the sense that he wants to hurt somebody. In a sexual way. You know. He’ll want a girlfriend or something like that. He’ll go out and get one or try their best anyway. Sometimes it doesn’t work (laughs slightly). But a man is a predator for sex, isn’t he? (Murphy & Winder, 2016, p. 806).

My hormones were going insane. (Hipp et al., 2017, p. 86).

[He] really had sexual tension going on, then [he] would need to do something [and] having to stop instantly [is] very difficult to do. (Jeffrey & Barata, 2018, p. 93).

### Theme Four: “I’m Really Sorry”

This was the least common theme across the data. It appeared in the accounts of participants in 4 of the 14 articles (Brennan et al., 2018; Hipp et al., 2017; Polaschek et al., 2001; Scully & Marolla, 1984). In those articles, it was coded in participant accounts 10 times.

While the previous themes have emerged in one guise or another in previous research, this theme appears not to have been previously recognized in the literature. In this theme, perpetrators seem to acknowledge and take responsibility for their use of sexual violence and express apparent empathy toward the victim and remorse for their actions. For example:

I wish there was something I could do besides saying “I’m sorry, I’m sorry.” I live with it 24 hours a day and, sometimes, I wake up crying in the middle of the night because of it. (Scully & Marolla, 1984, p. 41).

Then I start analyzing what I’ve done, what I’ve actually done to her. What am I going to do now to repair what I’ve done? (Polaschek et al., 2001, p. 535).

However badly I feel about it, and I do feel fucking revolted about it, I can only imagine she feels worse. I would literally sell my soul to be able to take that night back. (Brennan et al., 2018, p. 400).

## Discussion

The four themes that we developed from these 14 articles provide a rare and revealing first-hand insight into the perceptions and attitudes of male perpetrators of sexual violence. These findings suggest that the perceptions and attitudes of male perpetrators of sexual violence are complex but also somewhat predictable.

Many of the perpetrators in the studies described attitudes or beliefs that aligned with more than one of the themes. Some of the perpetrator accounts reported in the studies showed perpetrators simultaneously exhibiting, or at least alternating between, seemingly contradictory themes. This is consistent with earlier research identifying that male perpetrators often deploy contradictory and ambiguous narratives (Lea & Auburn, 2001). Perhaps the most common contradiction was that certain perpetrators sought to simultaneously normalize or downplay sexual violence (“she deserved it”) while also rejecting sexual violence (“I’m a good guy,” “It was out of character”).

At the same time, there was remarkable commonality of themes across the four decades covered by the studies. The persistence of themes across time was striking, given that over that period, there have been significant social changes and increasing attention to and condemnation of sexual violence. Similarly, there was remarkable commonality of themes across different settings in cohorts of perpetrators who had perpetrated different forms of sexual violence with different degrees of severity. Thus, themes that emerged in cohorts of incarcerated sexual offenders, some of whom had been convicted of multiple rapes and sexual murders, also emerged in accounts of more subtle and “normalized” forms of sexual violence—such as verbal coercion—given by college students and anonymous users of online communities. This is consistent with existing research (Lisak & Roth, 1988, 1990; Schewe et al., 2009).

In other words, the perspectives of male perpetrators were to some extent predictable across settings. This was especially true of the two most common themes: “she deserved it” and “I’m a good guy.” This is broadly supportive of the feminist structural analysis of sexual violence, which suggests that the prime contributor to such violence is not a perpetrator’s individual idiosyncrasies or pathologies but rather the structural and cultural influence of the patriarchal society in which perpetrators exist (Lea & Auburn, 2001; Phipps & Young, 2015). Perpetrators exist within and exemplify a “rape culture” in which sexual dominance is valorized and sexual violence is normalized (Hipp et al., 2017; Jeffrey & Barata, 2018; Scully & Marolla, 1985).

Relatedly, many of the themes echoed “rape myths” associated with patriarchal masculinity (Lea & Auburn, 2001; Sikweyiya et al., 2007, 2020). Implicit in many of the themes were the allied notions that (a) the use of sexual violence by men is normal, expected, and a reasonable form of punishment; and (b) that women are objects and an appropriate vessel into which to release male sexual desire. These myths infused, for example, the “she deserved it” and “I couldn’t stop myself” themes.

Another common strand of several themes was the perpetrator seeking to absolve himself of responsibility by displacing blame on to the victim or forces beyond his control. The displacement of blame was central to, for example, the “she deserved it,” “I’m a good guy,” and the “I couldn’t stop myself” themes. The displacement of blame seemed to be linked with a concern to avoid the label of “rapist” or equivalent. This was done by, for example, attributing sexual violence to an external cause, pathology, or an out of character event. Blame was variously displaced onto the victim, intoxicants, mental health or other life difficulties, and the male biology. In some cases, these justifications were presented in combination. For example, it was common for blame to be assigned to a combination of (a) the victim’s sexual provocation, (b) the disinhibiting effects of intoxicants, and (c) the perpetrator’s will being overborne by his sexual urges past a certain point of arousal. Again, this is consistent with previous research regarding denial and minimization of perpetrators (Kennedy & Grubin, 1992; Rogers & Dickey, 1991).

In most cases, perpetrators who sought to displace blame at all sought to displace blame completely. But the exception was the “I’m a good guy” theme. In that theme, perpetrators did not always seek to displace blame entirely but rather re-establish distance from their actions by explaining themselves. There was often some acceptance of blame accompanied by an attempt to reduce the perpetrator’s responsibility by appealing to mitigating factors: external circumstances (such as intoxicant abuse, mental illness, and life pressures) and the perpetrator’s good character that reveal their core and “stable” self (Ross, 1989). Strangely, some of these perpetrators perceived (and presented) evidence of their good character derived from their account of the sexual violence for which they accepted some responsibility. This included, for example, perpetrators who emphasized their attention to the comforts and sexual needs of their victim(s). As has been seen, the “I’m a good guy” theme was one of only two themes which accommodated some expression of regret or consideration for the victim.

The other theme which accommodated such expression was the least common but perhaps the most promising of the four themes: the “I’m really sorry” theme. This theme involved acknowledgment of and responsibility for the perpetrators’ use of sexual violence and expressions of genuine feelings of empathy for the victim and remorse. This is a particularly significant theme as it has not previously been identified in the literature. While it is not a particularly pervasive theme, it warrants further exploration at least in part because it may reveal something about what gives rise to remorse and the acceptance of responsibility and how the development of remorse and responsibility may be inculcated in intervention or rehabilitation programs. These critical findings are summarized in Table 3 below.

**Table 3.** Critical Findings.

1. Perceptions and attitudes of male perpetrators of sexual violence are complex. 2. No single perspective was universal; most perpetrators exhibited multiple perspectives, even contradictory perspectives. 3. There was remarkable commonality of perspectives across time and different settings in cohorts of perpetrators (incarcerated sexual offenders, college students and anonymous users online) who had perpetrated different forms of sexual violence with very different degrees of severity. 4. The commonality of perspectives suggests that a prime contributor to sexual violence is the structural and cultural influence of the patriarchal society in which most perpetrators exist. 5. Many of the most common perspectives shared underpinnings associated with rape myths. 6. A feature of the most dominant perspectives is the displacement of blame onto some combination of the victim, intoxicants, mental health or other life difficulties, and male biology. 7. Only the two least common perspectives accommodated some expression of regret or consideration for the victim. 8. The least common of these perspectives—“I’m really sorry”—is the most promising because it involved some responsibility, remorse, and empathy.

## Limitations and Implications

This scoping review reveals how little we know about the perceptions of male perpetrators of sexual violence with respect to their offending. The body of relevant peer-reviewed research is very narrow, and much of it is dated. The most comprehensive studies are those involving incarcerated sexual offenders in the late 1980s. The most significant and influential study, which produced three of the 14 articles and involved the largest cohort by some margin, is now nearly 40 years old (Scully & Marolla, 1985). Moreover, with some recent exceptions, the evidence is skewed toward serious sexual offenders in a custodial setting and sexual violence perpetrated against strangers. This is significant limitation given that most sexual violence is perpetrated by someone known to the victim and goes largely unreported. Intimate partner sexual violence, for example, is both prevalent and harmful, yet only one study focused on this form of sexual violence. Similarly, despite the over-representation of young men among perpetrators, only the two college campus studies specifically focused on this cohort.

There was a lack of diversity across the studies, with all but one being conducted in English-speaking, high-income countries. This is problematic given the worldwide prevalence of sexual violence and the particularly high rates reported in many low-and-middle-income countries (Borumandnia et al., 2020; World Health Organization, 2021). We recognize that cultural attitudes toward sexual violence, as well as socially accepted expressions of masculinity and norms around sexual consent navigation, vary enormously between countries and contexts. The perceptions, motivations, and understandings identified in this review may, therefore, be less applicable to men living in countries not represented within the included studies. Indeed, research with victims of sexual violence strongly supports the interaction of sociocultural factors such as family dynamics, religious and community values, globalization and poverty with the lived experience of sexual violence (Abeid et al., 2014; Lewis et al., 2020; Tummala-Narra et al., 2023). It is critical that this intersectional understanding be similarly developed for the perpetrator context.

Our review confirms the need for and identifies areas to target in future research. While the research indicated some commonality of perspectives across some different cohorts of perpetrators, the extent of that commonality remains somewhat uncertain. There remains a need—an urgent need—for multi-disciplinary, perpetrator-centered research charting the perspectives of understudied but relevant perpetrator cohorts. Specifically, further research is needed with respect to:

• male perpetrators of sexual violence against an intimate partner or other non-stranger, such as a friend and acquaintance;
• young male perpetrators of sexual violence; and
• male perpetrators from different cultural backgrounds and countries outside the “Global North.”

Such research is needed not only to understand these cohorts but also the extent to which common cultural, structural, and social factors supply or shape the perspectives and justifications of disparate perpetrator cohorts. Additionally, this review—and in particular the final theme—indicates a new line of enquiry. It suggests that there is at least a small subset of perpetrators who exhibit some level of empathy and remorse. There is a need for further research to explore this phenomenon and to understand how and why it arises, and if possible, how it can be encouraged.

Our analysis also highlights how stigmatized and complex this issue is and the extent to which male perpetrators seek to avoid the shame and ostracization associated with the label of “sex offender” or “rapist” (Sikweyiya et al., 2007, 2020). Many perpetrators expend significant psychological and narrative resources to try and avoid this label and protect their identity by displacing or mitigating blame. This points to the importance of nuanced methodological approaches (Stutterheim & Ratcliffe, 2021) in future research. This is particularly so where qualitative research is conducted in an in-person or at least a non-anonymous setting (Sikweyiya et al., 2007). There is a need for a degree of epistemic humility in such settings. It is imperative that researchers and interviewers are cognizant of their own assumptions, vulnerabilities, and shame triggers when approaching these topics (Sikweyiya et al., 2007). This will be necessary to hold safe and non-judgmental spaces where men can share their stories truthfully (Waldram, 2007), and wrestle with this simultaneously normalized and stigmatized form of violence. Otherwise, there is a risk that male participants will self-censor or alter their accounts to avoid anticipated stigma (Shaw, 2005). These implications are summarized in Table 4 above.

**Table 4.** Implications for Practice, Policy and Research.

1. The existing research is narrow, dated, and skewed toward serious sexual offenders who have offended against strangers and are incarcerated. 2. Further research is needed in respect of: (a) male perpetrators of intimate partner sexual violence, or at least non-stranger sexual violence; (b) young male perpetrators of sexual violence; and (c) male perpetrators from diverse cultural backgrounds and countries outside the “global North.” 3. The research suggested possible topics for further investigation, including perpetrator remorse and empathy. 4. There is significant stigma attached to the issue of sexual violence, requiring nuance and epistemic humility on the part of the researcher, as well as an awareness of the researcher’s own assumptions and shame triggers in respect of male perpetrators.

## Conclusion

Research on the perceptions and attitudes of male perpetrators of sexual violence remains limited and dated. This scoping review provides some understanding of perpetrators of sexual violence attitudes and perceptions, as well as directions for future perpetrator-centered research. It also revealed that the perceptions and attitudes of male perpetrators of sexual violence are complex and to some extent, predictable across settings, which suggest the powerful influence of structural and cultural factors in a patriarchal society. In order to comprehensively understand this issue, it must be studied in a multi-disciplinary way.

## Notes

1. We use the word “victim” for reasons of simplicity and consistency with the terminology used in the articles discussed, and without intending any disrespect to victim/survivors.
